# A common variant of the MACC1 gene is significantly associated with overall survival in colorectal cancer patients

**DOI:** 10.1186/1471-2407-12-20

**Published:** 2012-01-17

**Authors:** Alois H Lang, Simone Geller-Rhomberg, Thomas Winder, Nicole Stark, Klaus Gasser, Bernd Hartmann, Bertram Kohler, Ina Grizelj, Heinz Drexel, Axel Muendlein

**Affiliations:** 1 Vorarlberg Institute for Vascular Investigation and Treatment, A-6800 Feldkirch, Austria; 2 Department of Medicine and Cardiology, Academic Teaching Hospital Feldkirch, A-6800 Feldkirch, Austria; 3 Private University of the Principality of Liechtenstein, FL-9495 Triesen, Principality of Liechtenstein; 4 Drexel University College of Medicine, Philadelphia, PA 19104, USA

## Abstract

**Background:**

The newly discovered metastasis-associated in colon cancer-1 (MACC1) gene is a key regulator of the HGF/MET pathway. Deregulation of HGF/MET signaling is reported as a prognostic marker for tumorigenesis, early stage invasion, and metastasis. High expression levels of MACC1 have been associated with colon cancer metastasis and reduced survival. Potential links between the genetic diversity of the MACC1 locus and overall survival are unknown. We therefore investigated the association between MACC1 tagging single nucleotide polymorphisms (SNPs) and overall survival in a large cohort of colorectal cancer patients.

**Methods:**

The study included 318 subjects with histopathologically proven colorectal cancer at the Academic Teaching Hospital Feldkirch, Austria. Survival data were provided by the federal agency for statistics in Austria. Genomic DNA was isolated from formalin-fixed paraffin-embedded specimens; six tagging SNPs (rs1990172, rs3114446, rs10275612, rs3095007, rs3095009, and rs7780032), capturing most of the common variants of the MACC1 locus, were genotyped by SNaPshot assays.

**Results:**

Over a mean follow up period of 5.3 (± 1.0) years, 94 deaths were recorded. Carriers of the G-allele of SNP rs1990172 showed a significantly decreased overall survival (additive HR = 1.38 [1.05-1.82]; *p *= 0.023). Multivariate analysis adjusted for age and UICC tumor stage confirmed this result (HR = 1.49 [1.12-1.98]; *p *= 0.007). Other investigated genetic variants of the MACC1 gene were not significantly associated with overall survival (*p*-values > 0.05).

**Conclusions:**

For the first time, our study investigated the influence of MACC1 tagging polymorphisms on overall survival suggesting SNP rs1990172 as a predictor for reduced overall survival in colorectal cancer patients. Further studies will be required to validate our findings.

## Background

Colorectal cancer (CRC) is one of the most frequent malignancies in the Western world and one of the leading causes of cancer related deaths [1,2]. Metastatic dissemination of primary tumors is directly linked to patient's survival and accounts for about 90% of all CRC deaths [3]. Local invasion and the formation of metastases are clinically the most relevant processes involved in carcinogenesis, but their molecular mechanisms are not fully understood. There is growing evidence that the genetic heterogeneity of CRC has a major influence on its prognosis and the search for adequate molecular prognostic markers has come into focus of translational cancer research.

A significant success in this effort has been the identification of the metastasis-associated in colon cancer-1 (MACC1) gene as a crucial prognostic factor for CRC metastasis, as recently reported by Stein and colleagues [4]. The previously undescribed gene MACC1 (formerly designated as 7a5) was discovered by a genome-wide search for differently expressed genes in human colon cancer tissues, metastases, and normal tissues. In this study, high expression levels of MACC1 correlates positively with colon cancer metastasis and reduced metastasis-free survival [4]. Further, subsequent studies have shown that overexpression of MACC1 is associated with poor disease-free survival in patients with gastric carcinoma [5] and lung adenocarcinoma [6], respectively, and it was found that MACC1 is more frequently expressed in vascular invasive hepatocellular carcinoma [7]. These findings suggest that MACC1 may serve as a new parameter for the prognostic prediction of different kinds of cancer.

MACC1 acts as a master regulator of HGF-MET signaling pathway [4,8], whose activation has been found to play a critical role in oncogenesis and cancer metastasis [9,10]. Consequently, MET overexpression has been associated with poor clinical outcome [11,12]. Clinical studies of the therapeutic efficacy of MET-inhibitors in metastatic CRC are ongoing. Of note, it has been shown that overexpression of MACC1 correlates better with unfavourable pathologic features than overexpression of MET. Moreover, bioinformatic analysis of putative MACC1 targets identified elements besides MET, whose overexpression cosegregated with aggressive forms of CRC [13]. These data indicate that MACC1 could contribute to CRC progression through mechanisms other than or additional to MET transcriptional upregulation.

The MACC1 gene is located on chromosome 7 at position 7p21.1. Gain of chromosome 7 or selective gain of the p-arm is a relatively frequent occurrence in CRCs [13,14]. These chromosomal alterations might provide a link to the mechanisms leading to induced MACC1 expression. Further, numerous single nucleotide polymorphisms (SNPs) have been discovered in the human MACC1 gene (http://www.ncbi.nlm.nih.gov/projects/SNP). Some of these genetic variants may also contribute to altered MACC1 expression or function and as a consequence influence the prognosis of CRC. However, the impact of MACC1 SNPs on the clinical outcome of CRC has not been investigated yet.

We therefore investigated the association between MACC1 tagging SNPs capturing the majority of common alleles at the MACC1 locus and overall survival in a large cohort of colorectal cancer patients.

## Methods

### Patients

The present study included 318 white patients with histologically proven colorectal cancer diagnosed at the Department of Pathology at the Academic Teaching Hospital Feldkirch, Austria, from January 2003 to October 2006. All tumours were graded by an experienced pathologist using 6th edition of UICC classification [15]. After appropriate investigational review board approval, formalin fixed, paraffin embedded (FFPE) tissue blocks were recovered. Follow-up survival data were provided by the Federal Agency for Statistics in Austria. The Ethics Committee of the Land Vorarlberg, Austria, approved the present study.

### SNP Selection

**Figure 1:**
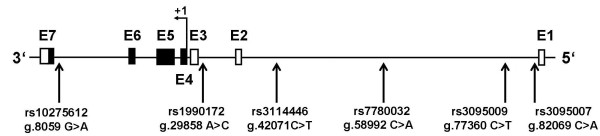
**Genomic positions of selected MACC1 tagging SNPs**. MACC1 is located on the minus strand of chromosome 7. The MACC1 contains seven exons (E1-E7) and six introns. Coding regions are shown as black boxes and non-coding exons as white boxes. Positions of the genotyped MACC1 SNPs are relative to the first nucleotide of the MACC1 gene as given in the NCBI reference sequence NT_079592.2. All selected SNPs are located in intronic regions.

Six tagging SNPs (rs1990172, rs3114446, rs10275612, rs3095007, rs3095009, and rs7780032) were selected from the HapMap SNP database [16], release #27; analysis panel: CEU + TSI (Utah residents with ancestry from Northern and Western Europe as well as Tuscan residents in Italy), using as criteria a minor allele frequency (MAF) ≥ 0.15 and pairwise r^2 ^≥ 0.8 according to Tagger software [17] implemented in Haploview program [18]. According to HapMap SNP database, these tagging SNPs capture 75% of variants with a MAF ≥ 0.15 within a region comprising 94 kb of chromosome 7, position 20,140,000 to 20,234,000 (NCBI build 36, hg18), including approximately 11 kb 5'-flanking and 6 kb 3'-flanking sequences of the MACC1 gene. The genomic positions of selected MACC1 SNPs are shown in Figure 1. All six tagging SNPs are located in intronic regions.

### Genotyping

Genomic DNA was extracted from deparaffinized FFPE tissue samples using the peqGOLD^® ^Tissue DNA Mini Kit (PEQLAB Biotechnologie Ltd., Erlangen, Germany) according to the manufacturer's instructions. Genotyping of all selected SNPs was carried out by the SNaPshot^® ^method (Applied Biosystems, Forster City, CA) consisting of PCR and multiplexed single-base extension, followed by capillary electrophoresis, as described in Additional file 1 ("Genotyping of MACC1 SNPs by the SNaPshot^® ^method"). In brief, genetic regions flanking the SNP of interest were amplified by PCR. PCR-products were used in a multiplex single-base primer extension reaction using the SNaPshot Multiplex Kit (Applied Biosystems), according to the manufacturer's protocol. PCR primer sequences and extension primer sequences are given in Additional file 2: Table S1 and Additional file 3: Table S2 respectively. Finally, labeled extension products were resolved by capillary electrophoresis on an ABI 3130 DNA Analyzer (Applied Biosystems) and data analysis was performed using GeneMapper Analysis Software version 4.0 (Applied Biosystems). Further, genotyping of 20 randomly selected samples was performed by Sanger sequencing to assess quality of SNP genotyping.

### Statistical analysis

Overall survival time was calculated from the time of histopathological diagnosis to death from any cause or the last follow up, at which point survival data were collected. Hazard ratios (HR) and 95% confidence intervals (CI) of the hazard ratios were derived from univariate and multivariate Cox proportional hazards models. Survival curves were generated using the Kaplan-Meier method and compared using the Log-Rank-Mantel-Cox-Test. Observed numbers of each genotype were compared with those expected to test whether the sample was in Hardy-Weinberg equilibrium using the Chi-Square test with one degree of freedom. To measure linkage disequilibrium, the squared correlation coefficient r^2 ^was calculated for each pair of SNPs using CubeX software (http://www.oege.org/software/cubex[19]). *P*-values < 0.05 were considered as significant. Statistical analyses were performed with the software package SPSS 11.5 for Windows (SPSS, Inc., Chicago, IL, USA).

## Results

### Patient characteristics

Over a mean follow up period of 5.3 (SD ± 1.0) years, 94 deaths were recorded, thus 29.6% of the patients died. The patient characteristics at baseline with respect to overall survival are presented in Table 1. Age at cancer diagnosis and UICC stage were significantly associated with overall survival.

**Table 1:** Patient characteristics with respect to overall survival

			Overall survival	
		**n**	**HR (95% CI)**	***p*-value**

Age	<60	62	1	
	60-70	104	2.28 [0.99-5.27]	0.054
	>70	152	2.09 [1.41-3.09]	<0.001
Gender	Female	141	1	
	Male	177	1.21 [0.80-1.83]	0.357
UICC stage	Stage I	64	1	
	Stage II	128	2.15 [1.03-4.47]	0.041
	Stage III	97	1.67 [1.16-2.42]	0.006
	Stage IV	29	1.81 [1.30-2.37]	<0.001
Grade score*	G1	93	1	
	G2	193	1.09 [0.69-1.71]	0.723
	G3-4	28	1.14 [0.78-1.67]	0.504
Tumor localisation **	Colon	219	1	
	Rectal	88	1.39 [0.91-2.13]	0.127

*Grade score, missing samples: n = 4; Tumor localisation, missing samples: n = 11

Hazard ratios (HR) and 95% confidence intervals (95% CI) were obtained from univariate Cox regression analysis

### Results from genotyping

Genotypes were successfully called in 302 patients for SNP rs3095007 (95.0%), in 307 patients for SNP rs7780032 (96.5%), in 311 patients for SNP rs3095009 (97.8%), and in 318 patients for SNP rs3114446, rs1990172, and rs10275612 (100%), respectively. Results of re-genotyping analysis of 20 randomly selected samples were 100% in agreement with the initial genotyping results. Observed MAFs and genotyping frequencies as well as results from Hardy-Weinberg disequilibrium analysis are presented in Additional file 4: Table S3. MAFs were similar to those given in HapMap SNP database [16] for a CEU + TSI analysis panel and all six MACC1 SNP genotype frequencies did not deviate significantly from Hardy-Weinberg equilibrium. SNPs were in low linkage disequilibrium or did not show any linkage disequilibrium (r^2 ^ranging from 0.45 to 0, as presented in Additional file 5: Table S4); therefore, no further haplotype analysis was conducted.

### Association of determined MACC1 SNP genotypes with overall survival

**Figure 2:**
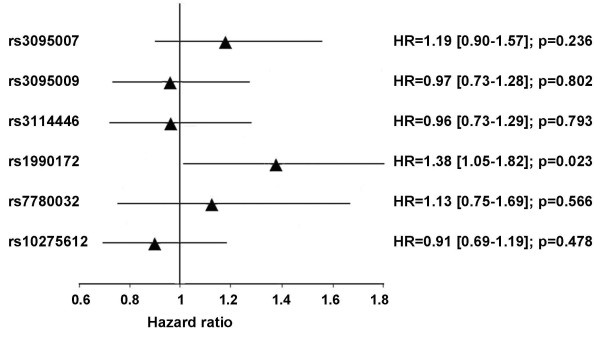
**Influence of determined MACC1 tagging SNPs on overall survival shown for an additive genetic model of inheritance**. Hazard ratios and 95% confidence intervals were obtained from univariate Cox regression analysis.

Impact of included MACC1 SNPs and overall survival was evaluated by univariate Cox regression analysis using an additive model of inheritance. Results are presented in Figure 2. Among investigated SNPs, variant rs1990172 was significantly associated with an increased risk for any death. Remaining SNPs of the MACC1 locus did not show a significant impact on overall survival.

The impact of variant rs1990172 on overall survival was further assessed by univariate and multivariate Cox regression analysis, adjusting for age und UICC stage, using an additive, dominant, and recessive model of inheritance, respectively. Results are presented in Table 2.

**Table 2:** Impact of MACC1 SNP rs1990172 on overall survival, shown for dominant, recessive, and additive genetic models of inheritance

Genetic model	Adjustment model	HR	95%CI	*P*-value
Additive	Model 1	1.38	1.05**-**1.82	0.023
	Model 2	1.49	1.12-1.98	0.007
Dominant	Model 1	1.59	1.05-2.40	0.028
	Model 2	1.63	1.08-2.47	0.020
Recessive	Model 1	1.50	0.86-2.60	0.152
	Model 2	1.82	1.04-3.18	0.036

Hazard ratios (HR) and 95% confidence intervals (95% CI) were obtained from univariate Cox regression analysis (model 1) and multivariate Cox regression analysis adjusting for age and UICC stage (model 2)

In an additive and dominant model of inheritance, carriers of the G-allele of SNP rs1990172 showed a significantly increased risk for reduced overall survival, in both, univariate and multivariate Cox regression analysis. A recessive model in univariate Cox regression analysis did not reach statistical significance, but became significant after adjustment for age und UICC stage.

**Figure 3:**
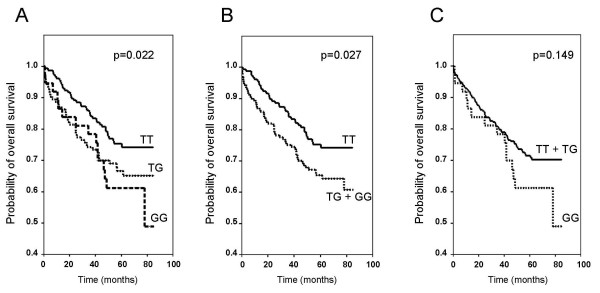
**Results from Kaplan-Meier analysis: Influence of MACC1 SNP rs1990172 on overall survival**. Kaplan-Meier curves are shown for an additive (**a**), dominant (**b**), and recessive model (**c**) of inheritance. *P*-values were calculated by Log-rank tests.

Additionally, Kaplan-Meier survival curves were graphically displayed according to an additive, dominant, and recessive model of inheritance (Figure 3). Again, SNP rs1990172 was significantly associated with overall survival under an additive and dominant model of inheritance.

## Discussion

In the present work, we report the novel finding of a positive association of the MACC1 tagging SNP rs1990172 with reduced overall survival in patients with CRC. To our best knowledge this is the first study investigating the influence of MACC1 polymorphisms on the prognosis of CRC. Several studies have investigated the influence of MACC1 mRNA levels on the clinical outcome in patients with colorectal [4], gastric [5] and lung cancer [6], respectively, providing evidence that MACC1 overexpression is a crucial prognostic factor for tumor recurrence, metastasis, and survival. Our finding that a tagging SNP in the MACC1 gene is significantly associated with patient's survival indicates that beside MACC1 mRNA levels also the genetic diversity of the MACC1 locus influences the prognosis of CRC. Therefore, our results emphasize the relevance of MACC1 as a prognostic marker also at the DNA level for clinical outcome prediction.

Overexpression of MACC1 may be caused by aneuploidy of chromosome 7, where MACC1 is located (position 7p21.1). Indeed, as shown by Galimi and colleagues [13], expression of MACC1 correlates with polysomy of chromosome 7 or with ploidy of the p-arm in metastatic CRC. However, in this study polysomy of chromosome 7 or ploidy of the p-arm mainly appeared at a low-level, with an average of 3.3 and 3.4 copies, respectively. Other, yet unknown molecular mechanisms may also contribute to MACC1 overexpression or function. SNPs located in coding or regulatory sequences have the potential to modify the biological activity of MACC1. Several putative deleterious SNPs are located at the MACC1 locus: One SNP leads to a premature stop codon at residue 390 (rs2108292) and two SNPs (rs36106647, rs35043094) shift the reading frame after amino acid 670, leading to a translational stop codon after 11 additional residues [20]. However, according to NCBI SNP database (http://www.ncbi.nlm.nih.gov/projects/SNP), these genetic variants are rare and will cause low statistical power, especially if penetrance is low, or even might be absent in a patient cohort of limited sample size. On the other hand, hundreds of common variants at the MACC1 locus of still unknown function are listed in the NCBI SNP database. Selection of a set of tagging SNPs based on stringent criteria like a high r^2 ^value allows covering most of this given genetic variance [21].

Here, we report the significant association of the MACC1 tagging SNP rs1990172 with reduced overall survival in CRC patients. Variant rs1990172 is located within an intronic region of the MACC1 gene (Figure 1) and does not affect any splice site of a coding exon. Therefore, it is unclear, if rs1990172 is the causative SNP responsible for the observed effects. Notably, the variant is in strong linkage disequilibrium with a non-synonymous SNP, rs975263 (r^2 ^= 0.858; http://www.hapmap.org), which is leading to an exchange of leucine to serine at codon 515, fifth exon. However, in silico analysis using software tools PolyPhen-2 (http://genetics.bwh.harvard.edu/pph2) [22] and SIFT (http://sift.jcvi.org) [23], respectively, does not suggest an adverse impact of this amino acid substitution on the structure or function of the MACC1 protein (data not shown). Both SNPs are located in a large haplotype spanning at least 50 kb of the MACC1 sequence, comprising three coding exons of the MACC1 gene. This indicates that also other SNPs in this haplotype may contribute to the prognosis of CRC.

Of note, according to data of HapMap recombination rates [24], two strong recombination hotspots outside the MACC1 locus around positions 20,091,000 and 20,607,000 (NCBI build 36) are defining a huge haplotype block including beside MACC1 another metastasis-associated gene, namely ITGB8 [25]. Therefore, an association between a SNP within the MACC1 locus and the clinical outcome might be linked to its correlation to causal genetic variants of the ITGB8 locus (position 20,337,000-20,420,000, NCBI build 36). However, linkage disequilibria between SNPs in the MACC1 and ITGB8 loci are at best of weak extend (e.g. r^2 ^values between rs1990172 and SNPs within the ITGB8 locus ranging from 0.00 up to 0.089; http://www.hapmap.org). Consequently, observed effect of the tagging SNP rs1990172 on overall survival appears predominantly caused by the genetic diversity of the MACC1 gene.

Genotyped SNPs rs3114446, rs10275612, rs3095007, rs3095009, and rs7780032 were not associated with overall survival. These variants are all located in non-coding or non-regulatory regions and are not in high linkage disequilibrium with any functional SNP given in HapMap SNP database [16]. Therefore, among investigated SNPs, variant rs1990172 remains the only one which is linked to a potentially functional region, which might explain the fact that among investigated SNPs solely rs1990172 is significantly associated with the prognosis of CRC.

In vitro studies have shown that MACC1 acts a key regulator of its transcriptional target gene MET [4,8], which encodes for the Met tyrosine kinase receptor for hepatocyte growth factor (HGF) [9]. Aberrant activation of MET deregulates the HGF/MET signaling pathway, leading to increased cell proliferation, invasion, and metastasis [9,10]. Consequently, high expression of MET in colorectal cancers is linked to the development of distant metastases and represents a strong prognostic indicator for poor survival [11,12]. These findings have led to the development of agents that can effectively disrupt HGF/MET signaling through direct inhibition of the receptor (anti-MET antibodies), through inactivation of its ligand HGF, by interfering with HGF binding to MET, or by inhibiting MET kinase activity [26]. Several phase I and II clinical trials addressing the therapeutic efficacy of these agents are currently under way. Amplification of the MET gene responds to Met inactivation with growth impairment gene in vitro and may therefore predict treatment outcome in vivo [13]. In a similar way, functional SNPs at the MACC1 locus associated with MACC1 activity or function may act as easily detectable predictive markers in anti-HGF/MET treatment in the future.

It is important to consider the potential limitations of our study. Overall survival was defined as the sole clinical end point evaluated. MACC1 is associated with colon cancer metastasis. Therefore, metastasis-free survival would represent a more accurate endpoint. However, the metastatic process is directly linked to patient survival [3] and, therefore, our observation that a MACC1 polymorphism is significantly associated with reduced overall survival also indicates its role in metastatic dissemination. Selected tagging SNPs captured 75% of variants with a MAF ≥ 0.15 and, therefore, 25% of common SNPs remained unexplored. Further, less frequent polymorphisms (MAF <0.15) were not considered by selection criteria. However, it is questionable if a probably moderate association between a polymorphism of low frequency and overall survival would have reached statistical significance due to given sample size. Further, it remains unclear, if the tagging variant rs1990172 or another variant, which is highly correlated with it, is the causative SNP responsible for the observed effects. Additional studies, such as fine mapping studies and directed functional studies to determine the molecular consequences of genetic variation at this locus are needed. Finally, we have not accounted for multiple testing in our study. Of note, the Bonferroni correction would probably have been too conservative owing to the given correlation among the tests performed. Therefore, associations between rs1990172 and clinical outcome appear significant only at a nominal significance level. Notably, the observed significant association between variant rs1990172 and overall survival would have survived Bonferroni correction in the adjusted additive model of inheritance (p_corrected _= 0.042). Replication of our observations in independent studies is necessary to clarify the prognostic relevance of MACC1 polymorphisms to the clinical course of CRC.

## Conclusion

Our study provides the first results that the genetic diversity of the MACC1 locus is associated with overall survival in colorectal cancer patients. The result of our study emphasizes the clinical relevance of MACC1 as a prognostic marker gene, which may help to select high-risk patients for more aggressive treatment strategies. Further studies are warranted to validate these findings.

## Competing interests

The authors declare that they have no competing interests.

## Authors' contributions

SGR, NS, and IG performed the experimental work. SGR and AM carried out data interpretation. AL, TW, and AM designed and coordinated the study. AL, SGR, and AM wrote the manuscript. TW, KG, NS, BH, BK, IG, and HD revised the manuscript critically for important intellectual content. All authors have read and approved the final manuscript.

## Pre-publication history

The pre-publication history for this paper can be accessed here:

http://www.biomedcentral.com/1471-2407/12/20/prepub

## Supplementary Material

Additional file 1 **Genotyping of MACC1 SNPs by the SNaPshot^® ^method**. Click here for file

Additional file 2 **Table S1**. MACC1 PCR primer sequences. Click here for file

Additional file 3 **Table S2**. MACC1 extension primer sequences. Click here for file

Additional file 4 **Table S3**. Observed minor allele frequencies and genotyping frequencies and results from Hardy-Weinberg disequilibrium analysis. Click here for file

Additional file 5 **Table S4**. Strength of pairwise linkage disequilibrium between each pair of genotyped SNPs expressed as r^2 ^and D'. Click here for file
