# Supplementary material for: A common variant of the MACC1 gene is significantly associated with overall survival in colorectal cancer patients
Source: BMC Cancer. 2012 Jan 17;12:20. doi: 10.1186/1471-2407-12-20 (PMC3282635; doi:10.1186/1471-2407-12-20)
Supplement: Additional file 1 — Genotyping of MACC1 SNPs by the SNaPshot® method. [file 1471-2407-12-20-S1.DOC]

**Supplemental text**: **Genotyping of MACC1 SNPs by the SNaPshot® method**

Genotyping of all selected SNPs was carried out by the SNaPshot® method (Applied Biosystems, Forster City, CA) consisting of PCR and multiplexed single-base extension, followed by capillary electrophoresis. First, genetic regions flanking the SNP of interest were amplified by PCR. PCR primer sequences are listed in supplemental table 1. PCR was performed in a 25 µl reaction volume mix, comprising 0.75 units AmpliTaq Gold® 360 DNA Polymerase (Applied Biosystems), 1x AmpliTaq Gold® 360 DNA Buffer (Applied Biosystems), 3 mM MgCl_2_, 0.6 µM of each Primer, 0.24 mM deoxynucleotide triphosphate and 5 µl genomic DNA of varying concentration under the following thermocycling conditions: 95°C for 10 min and 55 cycles of 95°C for 30 sec, 55°C for 30 sec and 72°C for 40 sec, followed by a last step at 72°C for 5 min. All six PCR products were pooled and 5 µl thereof treated with 2 μl of ExoSAP-IT® (GE Healthcare, Pittsburgh, PA) for 15 min at 37°C to remove unincorporated primers and dNTPs followed by 15 min at 80°C for enzyme inactivation.

The second step consisted of a multiplex single-base primer extension reaction using the SNaPshot Multiplex Kit (Applied Biosystems), according to the manufacturer’s protocol. Primer concentration in the 10 µl reaction mix was 0.2 µM of each extension primer. Extension primers consisted of a target specific sequence that ends one nucleotide 5'of the given SNP plus a poly(A) tail, which was added to ensure spatial resolution of the extension products during capillary electrophoresis. Extension primer sequences are given in supplemental table 2. During single-base extension reaction a fluorescently labeled ddNTP was added to each locus-specific extension primer. After the primer extension step, reactions were treated with 1 U of Calf Intestinal Phosphatase (NEB, Ipswich, MA) at 37°C for 60 min followed by 15 min at 75°C to prevent unincorporated fluorescent ddNTPs obscuring the primer extension products during electrophoresis.

Finally, labeled extension products were resolved by capillary electrophoresis on an ABI 3130 DNA Analyzer (Applied Biosystems) and data analysis was performed using GeneMapper Analysis Software version 4.0 (Applied Biosystems).
