# Supplementary material for: A common variant of the MACC1 gene is significantly associated with overall survival in colorectal cancer patients
Source: BMC Cancer. 2012 Jan 17;12:20. doi: 10.1186/1471-2407-12-20 (PMC3282635; doi:10.1186/1471-2407-12-20)
Supplement: Additional file 2 — Table S1. MACC1 PCR primer sequences. [file 1471-2407-12-20-S2.DOC]

**Supplemental table 1: MACC1 PCR primer sequences**

| Target | Primer | Sequence 5’ – 3’ |
| --- | --- | --- |
| rs3095007 | forward | CATCATGTATCATCTGTCTATTCAG |
|  | reverse | TGTGTGCATAACCTACCTCATT |
| rs3095009 | forward | CAATCTTATAGACTGTTGTTTATGGATATC |
|  | reverse | GGTTACCACTATCCTATCTTTGTCG |
| rs7780032 | forward | AGCTAAACTAAGGGAATGACTG |
|  | reverse | GTTTACCTTCCTAATGACATGTT |
| rs3114446 | forward | TAAAGAACTGTGTTACACCCATAC |
|  | reverse | CACAGCATAATGCCTGAGA |
| rs1990172 | forward | AAAGGAGGGAAGCATGTG |
|  | reverse | ATGCCACCTTATGAGACAATT |
| rs10275612 | forward | GTCAGACTCAATCTTGGTTGT |
|  | reverse | GGTAGTGTCCACGTCTCAGAG |
