# Supplementary material for: A common variant of the MACC1 gene is significantly associated with overall survival in colorectal cancer patients
Source: BMC Cancer. 2012 Jan 17;12:20. doi: 10.1186/1471-2407-12-20 (PMC3282635; doi:10.1186/1471-2407-12-20)
Supplement: Additional file 3 — Table S2. MACC1 extension primer sequences. [file 1471-2407-12-20-S3.DOC]

**Supplemental table 2: MACC1 extension primer sequences**

| Target | Sequence 5’ – 3’ | Lenght |
| --- | --- | --- |
| rs3095007 | aaaaaaaaaaaaaaaTGCACTGTGCTAATTTAATG | 35 |
| rs3095009 | aaaaaaaaaaaaaaaaaaaaaaCCACTGCTGTGTATGTGA | 40 |
| rs7780032 | aaaaaaaaaaaaaaaaaaaaaaaaaaaaaaaaaaaaaaaaaTTCCTGAGCTTTGGGCAT | 59 |
| rs3114446 | aaaaaaaaaaaaaaaaaaaaaTTTTATGGTTGAGTAATATTTCAC | 45 |
| rs1990172 | aaaaaaaaaaaaaaaaaaaaaaaaaaaaaaCCTTATGAGACAATTTTTGGAT | 52 |
| rs10275612 | aaaaaaaaaaaaaaaaaaaaaaaaaaaaaaaaaaaaaaaaaaaaaaaaaTCTTTGCAACTGAGCAG | 66 |

Poly(A) tails are indicated as lower cases.
