# Supplementary material for: A common variant of the MACC1 gene is significantly associated with overall survival in colorectal cancer patients
Source: BMC Cancer. 2012 Jan 17;12:20. doi: 10.1186/1471-2407-12-20 (PMC3282635; doi:10.1186/1471-2407-12-20)
Supplement: Additional file 4 — Table S3. Observed minor allele frequencies and genotyping frequencies and results from Hardy-Weinberg disequilibrium analysis. [file 1471-2407-12-20-S4.DOC]

**Supplemental table 3: Observed minor allele frequencies and genotyping frequencies and results from Hardy-Weinberg disequilibrium analysis**

| SNP | Base change (A>B) | MAF | Genotype frequency % (n) | | | Hardy-Weinberg equilibrium | |
| --- | --- | --- | --- | --- | --- | --- | --- |
|  |  |  | AA | AB | BB | *X*^2^ | p-value |
| rs3095007 | C>A | 0.296 | 51.7 (156) | 37.4 (113) | 10.9 (33) | 3.19 | 0.074 |
| rs3095009 | T>C | 0.457 | 30.5 (95) | 47.6 (148) | 21.9 (68) | 0.52 | 0.471 |
| rs3114446 | C>T | 0.378 | 39.6 (126) | 45.3 (144) | 15.1 (48) | 0.42 | 0.517 |
| rs1990172 | A>C | 0.311 | 49.4 (157) | 39.0 (124) | 11.6 (37) | 2.61 | 0.106 |
| rs7780032 | C>A | 0.126 | 77.5 (238) | 19.9 (61) | 2.6 (8) | 2.72 | 0.099 |
| rs10275612 | A>G | 0.434 | 34.6 (110) | 44.0 (140) | 21.4 (68) | 3.43 | 0.064 |

‘A’ refers to the common allele and ‘B’ refers to the minor allele in a white population; MAF refers to minor allele frequency; *X*^2^ refers to chi-squared.
