# Supplementary material for: A common variant of the MACC1 gene is significantly associated with overall survival in colorectal cancer patients
Source: BMC Cancer. 2012 Jan 17;12:20. doi: 10.1186/1471-2407-12-20 (PMC3282635; doi:10.1186/1471-2407-12-20)
Supplement: Additional file 5 — Table S4. Strength of pairwise linkage disequilibrium between each pair of genotyped SNPs expressed as r2 and D'. [file 1471-2407-12-20-S5.DOC]

**Supplemental table 4: Strength of pairwise linkage disequilibrium between each pair of genotyped SNPs expressed as r^2^ and D’.**

|  | | **D’** | | | | | |
| --- | --- | --- | --- | --- | --- | --- | --- |
|  |  | rs3095007 | rs3095009 | rs7780032 | rs3114446 | rs1990172 | rs10275612 |
| **r^2^** | rs3095007 |  | 0.76 | 0.77 | 0.77 | 0.49 | 0.46 |
|  | rs3095009 | 0.21 |  | 0.08 | 0.79 | 0.66 | 0.63 |
|  | rs7780032 | 0.04 | 0.00 |  | 0.79 | 0.63 | 0.75 |
|  | rs3114446 | 0.15 | 0.45 | 0.05 |  | 0.65 | 0.84 |
|  | rs1990172 | 0.05 | 0.23 | 0.03 | 0.30 |  | 0.85 |
|  | rs10275612 | 0.12 | 0.26 | 0.06 | 0.38 | 0.25 |  |
